# Supplementary material for: Surveillance for TB drug resistance using routine rapid diagnostic testing data: Methodological development and application in Brazil
Source: PLoS Comput Biol. 2024 Dec 23;20(12):e1012662. doi: 10.1371/journal.pcbi.1012662 (PMC11665995; doi:10.1371/journal.pcbi.1012662)
Supplement: S1 Table — (DOCX) [file pcbi.1012662.s001.docx]

# **SI Table 1. Descriptive associations between patient characteristics, selection into testing, and recorded rifampicin resistance**

|  | **New Cases** | | | | **Previously Treated Cases** | | | |
| --- | --- | --- | --- | --- | --- | --- | --- | --- |
| **Variable** | Tested with Xpert  (Odds) | | Resistant Xpert result  (Odds) | | Tested with Xpert  (Odds) | | Resistant Xpert result  (Odds) | |
|  | **(1)** | **(2)** | **(3)** | **(4)** | **(5)** | **(6)** | **(7)** | **(8)** |
|  | Univariate | Multivariate | Univariate | Multivariate | Univariate | Multivariate | Univariate | Multivariate |
| **Male** (Ref: Female) | 1.30 | 1.28 | 0.79 | 0.82 | 1.24 | 1.21 | 0.75 | 0.81 |
|  | (1.28, 1.31) | (1.27, 1.30) | (0.75, 0.84) | (0.77, 0.86) | (1.20, 1.27) | (1.18, 1.24) | (0.69, 0.81) | (0.75, 0.89) |
| **HIV Positive** (Ref: Negative) | 0.88 | 0.93 | 1.24 | 1.09 | 0.86 | 0.88 | 1.57 | 1.11 |
|  | (0.86, 0.89) | (0.91, 0.94) | (1.14, 1.36) | (0.99, 1.19) | (0.84, 0.89) | (0.86, 0.91) | (1.44, 1.72) | (1.01, 1.22) |
| **Age** (Ref: 25-34) | | | | | | | | |
| 0-4 | 0.25 | 0.28 | 1.82 | 1.53 | 0.23 | 0.24 | 2.16 | 1.72 |
|  | (0.23, 0.27) | (0.26, 0.30) | (1.30, 2.55) | (1.09, 2.15) | (0.17, 0.30) | (0.18, 0.32) | (0.92, 5.06) | (0.73, 4.05) |
| 5-14 | 0.38 | 0.42 | 1.88 | 1.64 | 0.32 | 0.34 | 2.37 | 2.03 |
|  | (0.36, 0.40) | (0.40, 0.44) | (1.51, 2.34) | (1.31, 2.04) | (0.26, 0.40) | (0.27, 0.42) | (1.26, 4.48) | (1.07, 3.85) |
| 15-24 | 0.99 | 0.99 | 1.02 | 1.02 | 0.96 | 0.96 | 0.96 | 0.98 |
|  | (0.97, 1.00) | (0.97, 1.01) | (0.94, 1.11) | (0.94, 1.11) | (0.92, 0.99) | (0.92, 0.99) | (0.84, 1.09) | (0.86, 1.12) |
| 35-44 | 0.87 | 0.89 | 1.19 | 1.15 | 0.99 | 1.00 | 1.14 | 1.05 |
|  | (0.86, 0.89) | (0.87, 0.90) | (1.10, 1.29) | (1.05, 1.24) | (0.96, 1.02) | (0.97, 1.03) | (1.03, 1.26) | (0.95,1.17) |
| 45-54 | 0.79 | 0.81 | 1.29 | 1.23 | 0.86 | 0.87 | 1.10 | 1.01 |
|  | (0.78, 0.81) | (0.79, 0.82) | (1.18, 1.40) | (1.13, 1.35) | (0.83, 0.89) | (0.84, 0.90) | (0.98, 1.24) | (0.90, 1.13) |
| 55-64 | 0.75 | 0.76 | 1.27 | 1.22 | 0.77 | 0.79 | 1.23 | 1.16 |
|  | (0.73, 0.76) | (0.75, 0.78) | (1.16, 1.40) | (1.11, 1.34) | (0.74, 0.80) | (0.75, 0.82) | (1.08, 1.41) | (1.01,1.33) |
| 65+ | 0.62 | 0.64 | 1.51 | 1.41 | 0.60 | 0.62 | 1.15 | 1.07 |
|  | (0.60, 0.63) | (0.63, 0.66) | (1.36, 1.67) | (1.28, 1.57) | (0.57, 0.63) | (0.59, 0.66) | (0.96, 1.38) | (0.89, 1.29) |
| **Level of the notifying health unit** (Ref: High complexity) | | | | | | | | |
| Low complexity | 1.68 | 1.58 | 0.62 | 0.65 | 1.46 | 1.36 | 0.43 | 0.45 |
|  | (1.66, 1.71) | (1.55, 1.60) | (0.57, 0.67) | (0.60, 0.71) | (1.41, 1.51) | (1.32, 1.41) | (0.39, 0.48) | (0.40, 0.50) |
| Medium complexity | 1.26 | 1.22 | 1.02 | 1.04 | 1.22 | 1.18 | 1.18 | 1.17 |
|  | (1.23, 1.28) | (1.20, 1.24) | (0.94, 1.10) | (0.95, 1.12) | (1.17, 1.26) | (1.13, 1.22) | (1.06, 1.32) | (1.05, 1.31) |
| Other | 0.66 | 0.63 | 0.76 | 0.80 | 0.57 | 0.55 | 0.54 | 0.56 |
|  | (0.63, 0.68) | (0.60, 0.65) | (0.62, 0.93) | (0.65, 0.98) | (0.53, 0.61) | (0.51, 0.59) | (0.40, 0.74) | (0.41, 0.77) |
| **Previously treated case type** (Ref: Re-entry) | | | | | | | | |
| Relapse | - | - | - | - | 0.91 | 0.94 | 0.87 | 0.95 |
|  |  |  |  |  | (0.89, 0.93) | (0.91, 0.96) | (0.81, 0.94) | (0.88, 1.03) |

# Odds with 95% confidence intervals in parentheses. Sample is restricted to TB notifications for new and previously treated cases between 2017-2023. This excludes individuals diagnosed with TB post-mortem, transferred to another facility, whose diagnosis type was unknown, and those who were misdiagnosed with TB. “Tested with Xpert” refers to the odds of receiving an Xpert test among all notified TB cases. “Resistant Xpert result” refers to the odds of having either a resistant or susceptible Xpert resistance result, among individuals who were tested with Xpert. This excludes individuals who were not tested with Xpert, who were not positive for TB with Xpert despite receiving a TB diagnosis, who had an indeterminant rifampicin result, and where the result was not recorded. For previously treated cases, “relapse” cases refer to individuals whose previous treatment outcome was cure. “Re-entry” cases are those who defaulted from treatment for at least 30 days. Level of the notifying health unit includes: Low complexity (first level of care), Medium complexity (second level of care, including TB referral networks), High complexity (tertiary level of care, including TB referral networks), and Other (e.g. lab, surveillance, clinics).
