# Supplementary material for: Surveillance for TB drug resistance using routine rapid diagnostic testing data: Methodological development and application in Brazil
Source: PLoS Comput Biol. 2024 Dec 23;20(12):e1012662. doi: 10.1371/journal.pcbi.1012662 (PMC11665995; doi:10.1371/journal.pcbi.1012662)
Supplement: Attachment — Submitted filename: Baum_PLOS Comp Bio_Revision_Reviewer Responses.docx [file pcbi.1012662.s002.docx]

## Reviewer #1:

Thank you for the opportunity of reviewing this interesting study about a key issue around TB. Drug resistance is a complex and serious problematic and being able to access accurate estimations is important for both research and local policy-making. This work has merit and I recommend its publication if the authors are able to address the points below.

Abstract
Lines 7-8: “RDT data collected in the course of routine (but not universal) use may provide biased estimates of resistance”
Unclear what the authors mean by ‘not universal’ in this context. Do they mean that RDT data are not collected in all countries? Or that RTD testing is not offered to all TB patients even within the same country? Please clarify and explain why the estimate may be biased.

Thank you for this comment. In lines 3-6, we have included a definition of what we mean by universal, meaning that not all patients are offered RDT testing: “In contexts where use of drug susceptibility testing has not been universal (i.e. not all patients are offered testing), surveillance for rifampicin-resistance—one of the core drugs in the TB treatment regimen—has relied on resource-intensive and infrequent nationally-representative prevalence surveys”.

We have also briefly mentioned that these estimates may be biased if the subset of patients that are tested do not represent the overall cohort of cases (lines 8-10): “However, RDT data collected in the course of routine (but not universal) use may provide biased estimates of resistance if the subset of patients receiving RDTs is not representative of the overall cohort”. As recommended further on, we have incorporated a longer discussion and examples of what these systematic differences entail (lines 68-71).

Lines 17-18: “Our estimates of RR-TB incidence were considerably more precise than WHO estimates for the same time period”
This is vague. The authors should clarify why they believe their estimates more precise, according to which metric.

We have edited this text to clarify that we are referring to model uncertainty, as quantified by the reported uncertainty intervals (lines 19-20): “Our estimates of RR-TB incidence were estimated with narrower uncertainty intervals relative to WHO estimates for the same time period, and were robust to alternative model specifications.” As the reviewer recommends below, we have briefly mentioned that the method uses recent case-level data from Brazil (lines 15-16) (“We applied this model to 2017-2023 case-level data on over 800,000 patients from Brazil.”) and have included a more in-depth explanation of differences in uncertainty in the discussion (lines 440-442) (“Our model estimates both the level and time trend from national case-level testing data on a much more recent time scale (2017-2023) with a sample of over 800,000 notified TB cases, which likely contributes to narrower uncertainty intervals.”).

Author Summary
Lines 30-32: “Applying this approach to Brazil, we find that modeled estimates were higher than naïve estimates, and were more precise compared to estimates produced by the World Health Organization.”
Same point as in the abstract, please clarify how these results are more precise than WHO’s estimates.

We have specified the use of recent case-level data as well as that we are referring to the uncertainty intervals being narrower (lines 39-41): “Applying this approach to recent national case-level data from Brazil, we find that modeled estimates were higher than naïve estimates, and with narrower uncertainty intervals compared to estimates produced by the World Health Organization.”

Introduction
Lines 56-57: “However, these data can yield biased estimates if RDT coverage is not universal, or there are systematic differences in access to testing.”
The authors should clarify what they mean by systematic differences in access to testing. Something systematic should be easier to estimate or predict, maybe they meant systemic differences? It would be good if they could provide examples.

We have provided some examples of why systematic differences in testing might arise (e.g. due geographic variation in access to RDTs, or if certain subsets of patients are more likely to be tested). Please refer to lines 68-71: “Systematic differences in testing may arise, for instance, if there is geographic variation in access to RDTs (e.g., urban or rural locations, proximity to health facilities or laboratories that perform testing) or if patients with certain observable characteristics are more likely to be offered testing.”

Lines 70-72: “countries that rely exclusively on national surveys to estimate prevalence among new cases accounted for almost 25.7% of the estimated annual number of incident TB cases”
Suggest rephrasing with the following for clarity: “countries that rely exclusively on national surveys to estimate drug resistance prevalence among new cases accounted for almost 25.7% of the estimated annual number of global incident TB cases”

Thank you. We have incorporated this change (lines 83-87): “Based on WHO estimates of TB incidence, countries that rely exclusively on national surveys to estimate TB drug resistance prevalence among new cases accounted for almost 25.7% of the estimated annual number of global incident TB cases, while 105 countries that exclusively rely on continuous surveillance accounted for 3.4% "

Method
Lines 170-184: This whole paragraph is very wordy. I appreciate the necessity to have a step-by-step explanation on how the statistical analysis is performed, but this should come with either formulas or a schematic, to make this visual and easier for the reader to follow. At the same time, the authors should go more into details on how WHO’s estimations are performed and how that method differs from their own, comparing the formulas/schematics.

We have reorganized the paragraph to more clearly outline our outcomes in lines 185-197. We have also included equations for the two modeled outcomes:

“Point estimates of rifampicin-resistance prevalence among notified TB cases for each area *j* at time *t* ($\mu_{jt}$) were calculated by summing non-missing resistance results and the imputed probabilities, divided by total TB notifications ($n_{jt})$ in area *j* at time *t* (1).

$$\mu_{jt}= \frac{\sum_{ijt}^{T_{i}=1} Y_{ijt}+ \sum_{i}^{T_{i}=0} \pi_{ijt}}{n_{jt}}$$

Total rifampicin-resistant notifications per 100,000 person-years in area *j* in quarter *t* ($\Psi_{jt}$) were calculated by dividing the modeled number of cases by the population ${(N}_{jt})$ in area *j* at quarter *t* (2).

$$\Psi_{jt}= \frac{4* \{\sum_{ijt}^{T_{i}=1} Y_{ijt}+ \sum_{ijt}^{T_{i}=0} \pi_{ijt}\}}{N_{jt}}* 100,000$$

We compared these estimates to those produced by a naïve approach assuming Xpert results were missing at random. The prevalence of rifampicin resistance was calculated as the share of conclusive Xpert resistance results that were resistant. Total RR-TB notifications per 100,000 person-years were calculated by dividing the number of resistant Xpert tests scaled by the fraction tested by the national or state-level population.”

Results
Lines 318-327: (1) The authors explain how their results differ from the WHO ones, however they fail to mention that estimations for latest years fit into WHO’s confidence intervals, which should be highlighted.

In lines 373-374, we have specified that for some years our estimates fall within the WHO’s uncertainty intervals: “For some years, our estimates fall within WHO’s uncertainty intervals.”

(2) The authors argue that WHO’s confidence intervals are wide, however I would argue that only the ones for previously treated cases are somewhat wide, while in new and total cases these are narrow, with the modelled uncertainty intervals being extremely narrow. The authors should address why the uncertainty in their estimations is so small.

Thank you for this comment. We’ve explained this in lines 439-442 of the discussion that we use recent, national case-level testing data with a large sample: “Our model estimates both the level and time trend from national case-level testing data on a much more recent time scale (2017-2023) with a sample of over 800,000 notified TB cases, which likely contributes to narrower uncertainty intervals.” We have also mentioned this in the abstract, as recommended by the reviewer (see above).

(3) Results also show a decreasing modelled trend compared to a somewhat constant trend in WHO estimations, however this difference seems to decrease as the model runs in recent years in new and total cases, while it seems to keep decreasing in previously treated. It would be good if the authors could expand on this either here or in the discussion.

We’ve added some additional text to the discussion in Lines 443-447 as to why this difference may exist: “Similarly, differences in estimation approach and input data likely explain the differences in the time trends estimated in our analysis compared to the WHO’s estimates – while our results reflect declining time trends in RIF resistance in the routine reporting data, the WHO estimates reflect approximately flat time trends in data from neighboring countries.”

Discussion
The authors mention how WHO’s estimates for Brazil rely on results from national MDR-TB/RR-TB prevalence survey from 2006-2008 and estimate the time trend using data from neighbouring countries. While their model estimates both the level and time trend from case-level testing data from Brazil’s National TB Program on a much more recent time scale (2017-2023). This is the reason why they are justified in arguing that their results might be more precise and it should be mentioned much earlier in the paper including the abstract, even just a few words about this method using recent local data and leave the lengthier explanation to the discussion.

Per this comment and others outlined above, we have updated the abstract (lines 15-16, 19-20), author summary (lines 39-41), and discussion (lines 443-447) to emphasize our data and its contribution to our narrower uncertainty intervals.

Additionally, I have not seen any mention of the test accuracy being taken into account, can the authors please clarify whether this was the case and, if not, mention this in the limitations and discuss if and how their results might change if they did.

You are correct in that we did not account for the accuracy of the Xpert MTB/RIF results in our analysis, given the high sensitivity and specificity reported for this component of the Xpert MTB/RIF assay of the test. We have added this as a limitation in line 482: “Fifth, we do not adjust our estimates for sensitivity and specificity of GeneXpert.”

## Reviewer #2:

Dr Baum and colleagues present in their manuscript a novel method for estimating/correcting TB resistance trends by using a hierarchical generalized additive regression model applied to existing rapid diagnostics tests results. The manuscript is exceptionally clear in their explanation of the method, and I commend the authors for their clarity and transparency in the description of methods as well as recognition of limitations.

In my capacity of infectious disease epidemiologist, I will limit my comments to the impact of this method for the field and the potential limitations from the epidemiological point of view, and will leave the statistical details for other experts in these methods.

First I need to say that I did not find major problems with this manuscript, so my suggestions are only minor.

The method proposed by Baum and colleagues is a robust approach that leverages from existing (if imperfect) data on RDTs. The method is very relevant to the field of TB burden estimation at local and global level and as I read the paper I could see how this could have a large impact on how we understand the real burden of TB resistance globally. A couple of considerations and suggestions:

- Under-reporting correction assumptions: The assumption behind under reporting corrections is a very important one. Although we can't prove the contrary, it is mostly likely that the probability of resistance is not the same between notified and non-notified TB cases. Although the authors recognise how this can be a potential issue, it would be useful to understand how this could be tackled in the future and what are the implications for the current results.

Thank you for this comment. We have added a sentence to the discussion (lines 487-489) describing what the potential implications of differences in probability of resistance might be among notified vs. non-notified cases: “If the probability of resistance were higher (lower) among non-notified cases, then our results would underestimate (overestimate) RR-TB prevalence in the population.”

- Applicability in other contexts: The case of Brazil is very particular, as they have an under the threshold fraction of RDT use at point of diagnosis, but they have a large, rather complete surveillance system (SINAN, SISCEL etc) which allows for a statistical model like the one presented to be designed and analysed. The authors do recognise this in their discussion but I believe that one of the aims of this work beyond this publication should be to propose a path to better estimates of RR-TB trends from those made by WHO. It would be useful if the authors could more accurately point to the settings in which this method could actively help improve estimates and propose a path forward.

We’ve added additional text in lines 498-500. This complements some of the existing language we included on which settings this method may be most useful (i.e. those already with or working towards robust surveillance systems, and that are implementing Xpert and know how testing is allocated): “Further, the applicability of this approach to other contexts depends on having and/or establishing a strong data infrastructure, and so would be more useful with additional investments in surveillance systems.”
